# Supplementary material for: Insights into the Molecular Mechanisms of Eg5 Inhibition by (+)-Morelloflavone
Source: Pharmaceuticals (Basel). 2019 Apr 16;12(2):58. doi: 10.3390/ph12020058 (PMC6630617; doi:10.3390/ph12020058)
Supplement: Supplementary file 1 [file pharmaceuticals-12-00058-s001.pdf]

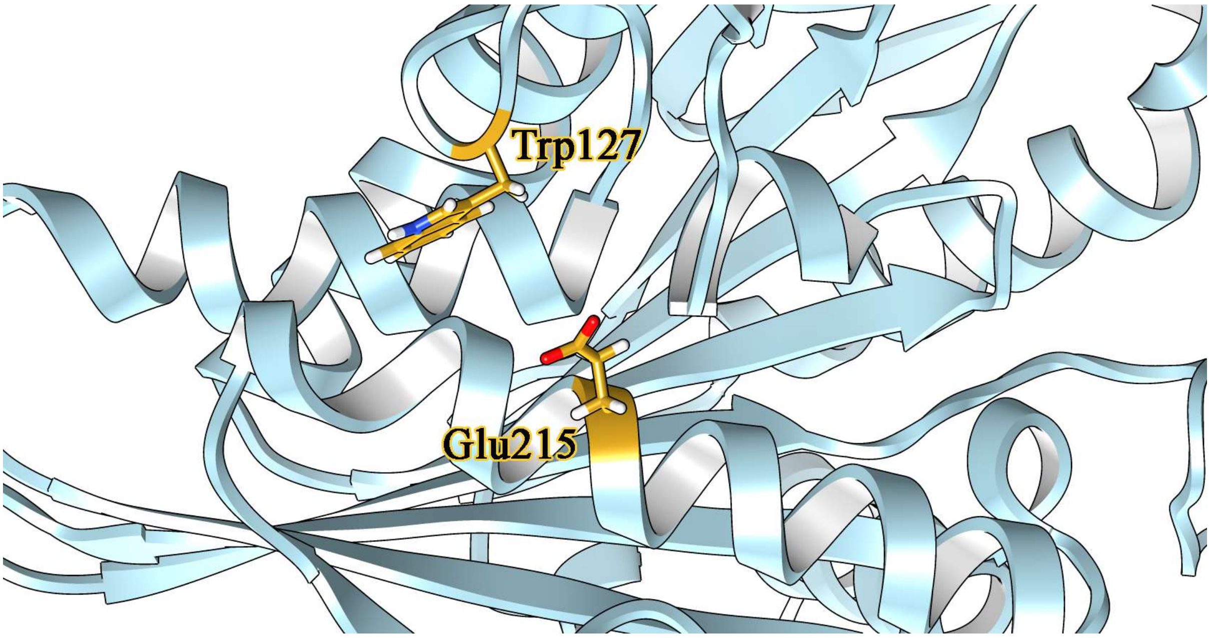

Figure S1. Cartoon representation of Eg5 crystal (motor domain) showing amino acid residues Trp127 and Glu215, shown as sticks (yellow).

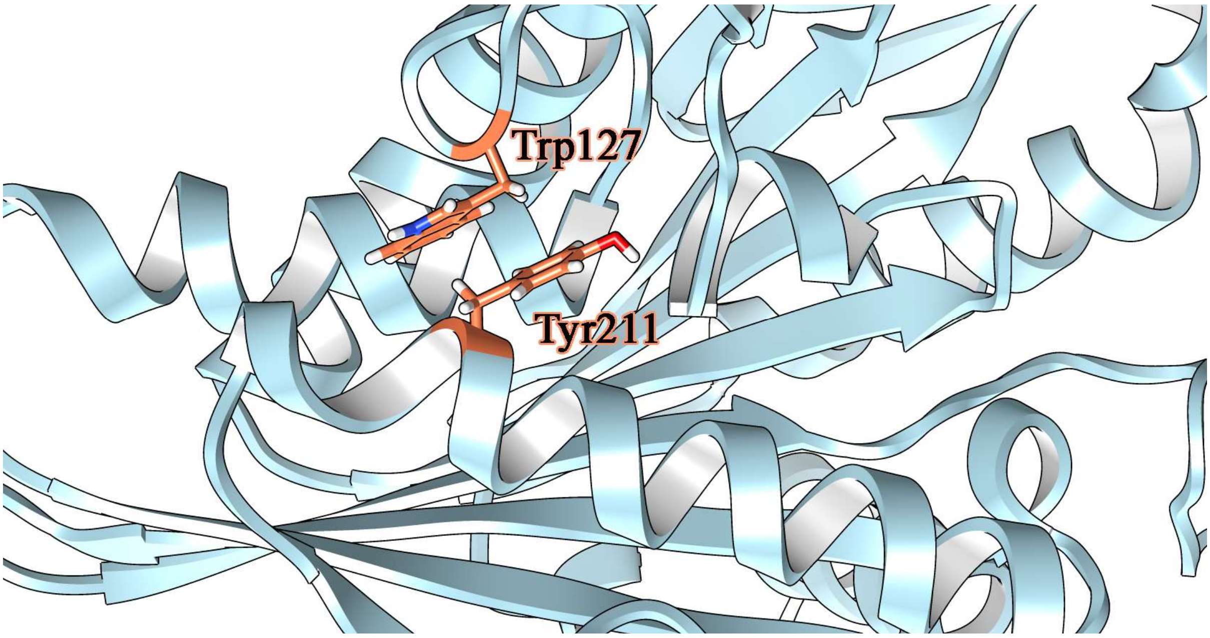

Figure S2. Cartoon representation of Eg5 crystal (motor domain) showing amino acid residues Trp127 and Tyr211, shown as sticks (orange).

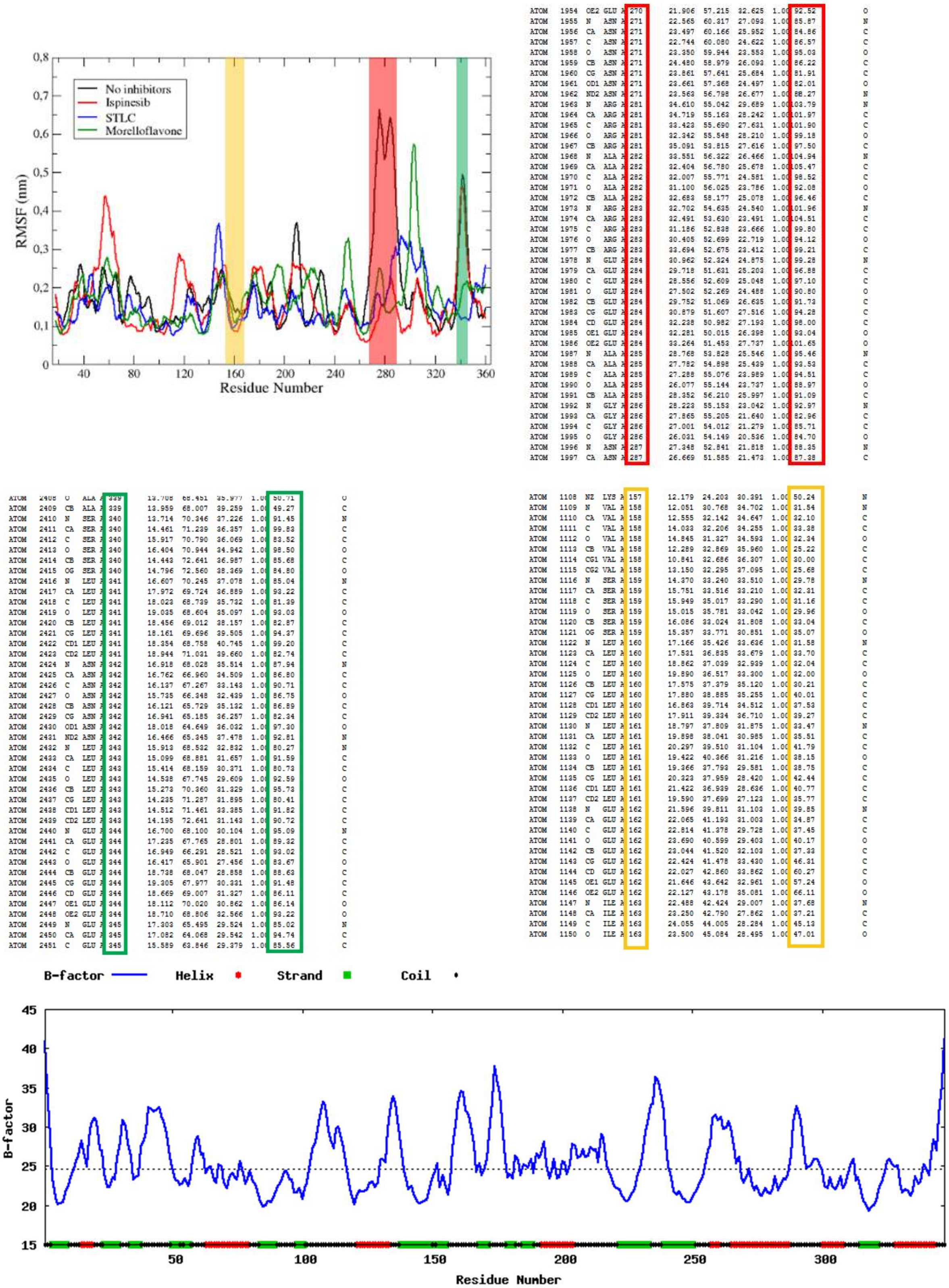

Figure S3. ***Comparison between RMSF and experimental b-factors.***

The simulations were validated by comparing the root mean square fluctuation (RMSF) of Eg5 residues with standardized crystallographic temperature factors (B-factors), which are a measure of the flexibility of the residues in the protein. These data are available on structural file and computed from experimental data. We compared the b-factor contained in the 4A5Y and 5ZO7 pdb files, which correspond to Eg5 in complex with ispinesib and STLC respectively, and we found that atoms with

higher values of flexibility compose the amino acids with the highest fluctuation degree. On the contrary, the atoms with the lower flexibility values found on the crystallographic temperature factor compose the amino acids with lowest fluctuation degree. These results confirmed the stability of the MD simulations. Using our Eg5 model, we also calculated b-factors for the apo protein on ResQ server (blue line). The plot obtained is compatible with the results from MD simulation.
